# Bonding of articular cartilage using a combination of biochemical degradation and surface cross-linking

**DOI:** 10.1186/ar2202

**Published:** 2007-05-15

**Authors:** Carsten Englert, Torsten Blunk, Rainer Müller, Sabine Schulze von Glasser, Julia Baumer, Johann Fierlbeck, Iris M Heid, Michael Nerlich, Joachim Hammer

**Affiliations:** 1 Department of Trauma Surgery, University Medical Centre Regensburg, Franz-Josef-Strauss-Allee, 93053 Regensburg, Germany; 2 Department of Pharmaceutical Technology, University of Regensburg, Universitätsstrasse, 93053 Regensburg, Germany; 3 Institute of Physical and Theoretical Chemistry, University of Regensburg, Universitätsstrasse, 93053 Regensburg, Germany; 4 Mechanical Engineering Faculty, University of Applied Sciences, Galgenbergstrasse, 93053 Regensburg, Germany; 5 GSF-National Research Centre, Institute of Epidemiology, Ingolstädter Landstrasse, 85674 Neuherberg, Germany; 6 Institute of Medical Informatics, Biometry, and Epidemiology, Ludwig-Maximilians-University, Munich, Germany

## Abstract

After trauma, articular cartilage often does not heal due to incomplete bonding of the fractured surfaces. In this study we investigated the ability of chemical cross-linkers to facilitate bonding of articular cartilage, either alone or in combination with a pre-treatment with surface-degrading agents. Articular cartilage blocks were harvested from the femoropatellar groove of bovine calves. Two cartilage blocks, either after pre-treatment or without, were assembled in a custom-designed chamber in partial apposition and subjected to cross-linking treatment. Subsequently, bonding of cartilage was measured as adhesive strength, that is, the maximum force at rupture of bonded cartilage blocks divided by the overlap area. In a first approach, bonding was investigated after treatment with cross-linking reagents only, employing glutaraldehyde, 1-ethyl-3-diaminopropyl-carbodiimide (EDC)/N-hydroxysuccinimide (NHS), genipin, or transglutaminase. Experiments were conducted with or without compression of the opposing surfaces. Compression during cross-linking strongly enhanced bonding, especially when applying EDC/NHS and glutaraldehyde. Therefore, all further experiments were performed under compressive conditions. Combinations of each of the four cross-linking agents with the degrading pre-treatments, pepsin, trypsin, and guanidine, led to distinct improvements in bonding compared to the use of cross-linkers alone. The highest values of adhesive strength were achieved employing combinations of pepsin or guanidine with EDC/NHS, and guanidine with glutaraldehyde. The release of extracellular matrix components, that is, glycosaminoglycans and total collagen, from cartilage blocks after pre-treatment was measured, but could not be directly correlated to the determined adhesive strength. Cytotoxicity was determined for all substances employed, that is, surface degrading agents and cross-linkers, using the resazurin assay. Taking the favourable cell vitality after treatment with pepsin and EDC/NHS and the cytotoxic effects of guanidine and glutaraldehyde into account, the combination of pepsin and EDC/NHS appeared to be the most advantageous treatment in this study. In conclusion, bonding of articular cartilage blocks was achieved by chemical fixation of their surface components using cross-linking reagents. Application of compressive forces and prior modulation of surface structures enhanced cartilage bonding significantly. Enzymatic treatment in combination with cross-linkers may represent a promising addition to current techniques for articular cartilage repair.

## Introduction

After trauma, articular cartilage often does not heal due to incomplete bonding of the fractured surfaces. The pathophysiological mechanism of articular cartilage integration has been intensively investigated *in vitro*, showing that integration depends on collagen metabolism [1,2], collagen cross-linking [3], cell vitality [4], and hormonal stimulation [5]. Inhibiting factors have also been described, such as synovial fluid components, which may inhibit the integrative repair by binding to the cracked surface [6], cytokines, which abolish the anabolic steroid hormone effect [5], and the synovial fluid flow, which might act at the interface in joint motion to keep the surfaces apart [7].

Based on these findings, therapeutic options for articular cartilage integration have been investigated. Collagen cross-linking has been stimulated over time *in vitro *[8] or articular cartilage surfaces have been degraded in order to stimulate repair *in vitro *[9-12] as well as *in vivo *[13]. Enzymes that were employed for articular cartilage degradation included trypsin [5,11,13], chondroitinase ABC [9], and hyaluronidase with subsequent collagenase treatment [12], resulting in enhanced integrative cartilage repair *in vitro*. Physical swelling of surface structures by guanidine was also reported to stimulate the integrative repair process [10].

Cartilage can be considered as a composite material consisting of a collagen network and other extracellular matrix components, mainly glycosaminoglycans (GAGs). Collagen and its derivatives have been cross-linked for tissue engineering or biomaterial purposes [14,15]. Glutaraldehyde is the most extensively used reagent for cross-linking primary amino groups, mainly exposed by collagen [16,17]. However, it has been reported to elicit cytotoxic effects [18,19]. In proteoglycans, amino groups are mainly acetylated and, therefore, not subjected to glutaraldehyde cross-linking. Water-soluble carbodiimides activate carboxylic groups of proteins such as collagen, which results in the formation of amide-type cross-links without any residual reactive groups [20,21]. In addition, carbodiimides were found to cross-link hyaluronic acid molecules by forming ester bonds between hydroxyl and carboxyl groups [22]. The carbodiimide method has been shown to be superior to glutaraldehyde in terms of cyto- and biocompatibility [19,23]. Another favourable cross-linker for primary amino groups is the naturally occurring reagent genipin, which has been reported to be significantly less cytotoxic than glutaraldehyde [23,24]. Transglutaminase, an enzyme in mammalian chondrocytes whose expression is strongly correlated with cell differentiation, has also been used as a collagen cross-linking reagent [25] and has been introduced for articular cartilage gluing [26]. Taken together, glutaraldehyde, carbodiimides, genipin, and transglutaminase all cross-link functional groups of extracellular matrix components. Such reagents may, therefore, also be used to cross-link exposed functional groups on a fractured surface of articular cartilage after trauma or transplantation.

The objective of this study was to investigate the initiation of immediate bonding of articular cartilage blocks by means of combining cartilage degradation and cross-linking reagents. In addition, it was investigated whether a considerable compression of the cartilage blocks was necessary to achieve bonding. For all combinations, that is, compression of cartilage blocks and application of surface degrading and cross-linking reagents, specific emphasis was put on the achievable adhesive strength of the bonding interface according to the integration model established by Reindel and colleagues [4].

## Materials and methods

### Cartilage preparation, compression, and bonding

**Figure 1:**
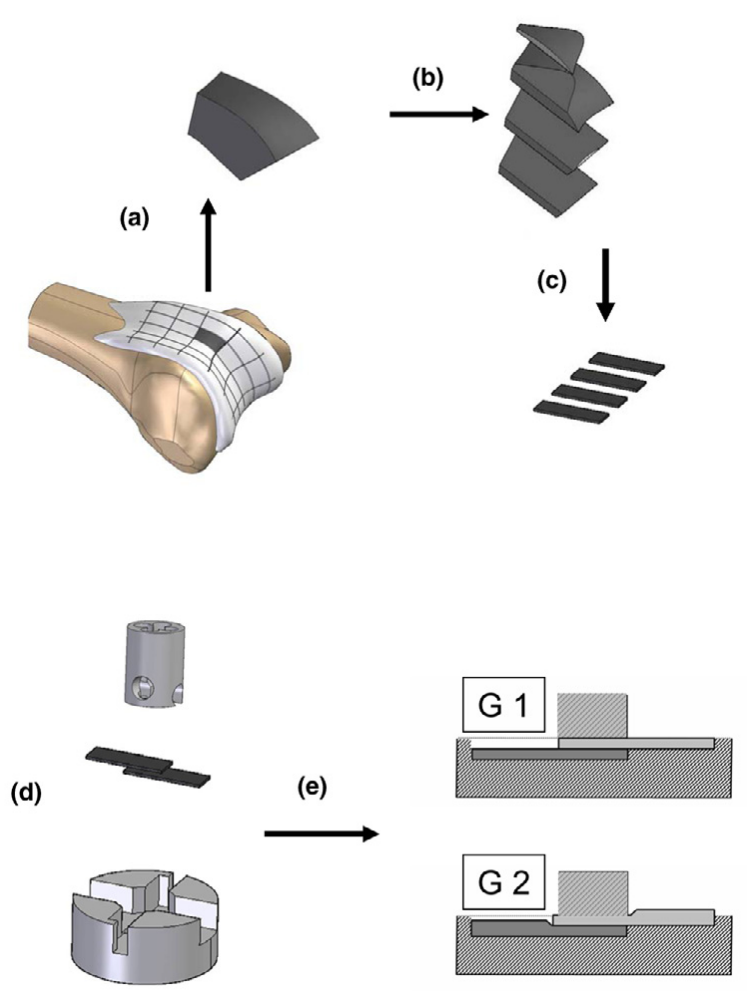
Preparation of the articular cartilage blocks and assembly during bonding experiments. **(a) **Osteochondral fragments were harvested from the femoropatellar groove of bovine calves. Blocks of 10 × 10 × 20 mm^3 ^were harvested. **(b) **These blocks were cut into cartilage slices of two precisely defined thicknesses, 0.25 and 0.3 mm, which were designated geometry 1 and 2 (G1 and G2) (in the diagram, only one thickness is shown). **(c) **These slices were cut into rectangles of 8 mm × 2.5 mm. **(d) **The integration specimens were assembled by positioning two cartilage rectangles in partial apposition, creating a defined overlap area of 4 mm × 2.5 mm for bonding. **(e) **When cartilage samples (either two G1 or two G2 blocks) were inserted into the chamber and fixed by the stamp, different compressive strains were applied.

Within one day after sacrificing of 8- to 12-week-old bovine calves, osteochondral fragments were harvested from the femoropatellar groove, using a reciprocating saw (Stryker Instruments, Kalamazoo, MI, USA). Blocks of 10 mm × 10 mm in length and 20 mm in height were harvested (Figure 1a). A sledge microtome (Microm HM440E, Neuss, Germany) was used to cut the osteochondral fragments into cartilage slices of two precisely defined thicknesses, that is, 0.25 mm (geometry one (G1)) and 0.3 mm (geometry two (G2)) (Figure 1b). In all cases, the two top slices were discarded and only the following two underlying slices were used for experiments. These slices were cut into rectangles of 8 mm × 2.5 mm (Figure 1c). During the entire preparation procedure the specimens were kept moist and free of blood by copious irrigation with cooled PBS.

The integration specimens were assembled by positioning two cartilage rectangles in partial apposition, creating a defined overlap area of 4 mm × 2.5 mm for bonding. The precise assembly was guaranteed by a custom-made chamber and an additional fixation stamp (Figure 1d) [5]. After fixation (without cartilage blocks) a gap of exactly 0.5 mm between stamp surface and chamber bottom remained. Thus, when the cartilage samples were inserted into the chamber and fixed by the stamp, for two G1 cartilage blocks almost no compression was acting during bonding, whereas for G2 blocks a defined compressive strain of 17% of the total thickness was applied (Figure 1e). To determine the creep modulus for both geometries (G1 and G2), the custom-made chamber and the stamp were modified and connected to the test rig (Hegewald and Peschke, Nossen, Germany). Samples were compressed by the stamp and the resulting force relaxation behaviour was analysed by recording the load over time.

### Cross-linking

**Figure 2:**
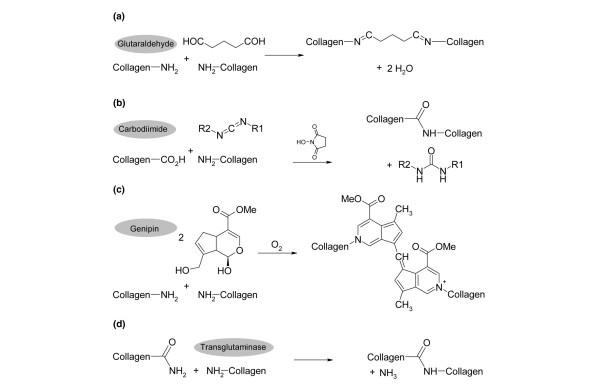
Schematic collagen cross-linking reactions for the employed reagents. **(a) **Glutaraldehyde covalently binds to amino groups, but can also bind to other glutaraldehyde molecules. **(b) **1-Ethyl-3-diaminopropyl-carbodiimide (EDC) and N-hydroxysuccinimide (NHS) catalyses covalent bindings between carboxylic acid and amino groups; thus, cross-linking between collagen structures is possible. Furthermore, other extracellular matrix components containing carboxyl groups, such as glycosaminoglycans, can also be cross-linked. **(c) **Genipin reacts in a similar manner as glutaraldehyde, but can only bind to one other genipin molecule. **(d) **Transglutaminase is a highly specific enzyme catalysing collagen cross-linking between lysine and glutamine in collagen structures with the release of ammonia.

For bonding, the cartilage blocks within the chamber were placed in a 24-well culture plate and each sample was subjected to one of the following cross-linking agents for 2 h at room temperature (750 μl per sample): glutaraldehyde (Roth, Germany) at a concentration of 20 mg/ml, buffered in PBS; 1-ethyl-3-diaminopropyl-carbodiimide (EDC) and N-hydroxysuccinimide (NHS) (Fluka, Neu-Ulm, Germany) at concentrations of 20 mg/ml and 5 mg/ml, respectively, in morpholinoethanesulfonic acid buffered solution (pH 5.5); genipin (WAKO Chemicals, Germany) at a concentration of 5 mg/ml in PBS; transglutaminase (Ajinomoto Foods, Hamburg, Germany) at a final concentration of 60 U per gram dry weight of cartilage block in 0.01 M acetic acid, adjusted to pH 6 (transglutaminase was applied according to the protocol by Chen and colleagues [27], with the exception that cartilage blocks were incubated in treatment solution for 2 h in contrast to the described protocol with a treatment duration of 12 h). See also Figure 2 for chemical reaction schemes.

### Surface degradation

For improved cross-linking, additional surface degradation treatments prior to cross-linking were applied. In all cases, the tissue samples, that is, the single cartilage pieces, were incubated in a 100 μl solution. After surface degradation, all samples were washed three times with PBS before being inserted into the chamber described above for bonding experiments. The following three solutions were used for degradation: trypsin (Gibco, Eggenstein, Germany) at a concentration of 0.5 μg/ml in PBS, pH 7.4 for 30 minutes at room temperature [6]; pepsin from hog stomach with 3,348 U/mg (Sigma-Fluka, Steinheim Germany), used at 0.5 mg/ml in PBS for 30 minutes, pH 7.4 at room temperature; guanidinium hydrochloride (Sigma, Steinheim Germany) at a concentration of 4 mol/l for 10 minutes at 10°C in a solution that was prepared with sodium acetate and adjusted to pH 6.0 by hydrochloric acid [10].

### Biomechanical testing

The adhesive shear strength after cross-linking was investigated under uniaxial tensile loading, as first described by Reindel and colleagues [4]. All experiments were performed until rupture. Prior to mechanical testing the integrated interface area of each sample was determined by optical microscopy using the imaging software analySIS 3.1 (SZX12, Olympus, Hamburg, Germany). The samples were carefully removed from the incubation chambers and mounted into the fixings of the test rig (Hegewald and Peschke). Particular care was taken to exclude any influence resulting from misalignment in the orientation of the load axis to the neutral fibre of the interface area by using a biaxial positioning device with an accuracy of 0.01 mm. Both custom-designed fixings were equipped with a small vacuum drill hole for accurate adjustment. The final fixing of the samples was achieved by spring-loaded jaws. The gauge length (that is, free distance between the fixings) was 7 mm in all cases.

All tests were run at an extension rate of 0.5 mm/minute. The displacement was continuously measured as the increase in distance between the two fixings by means of a linear variable differential transformer with an accuracy of 0.01 mm (HBM, Inc., Marlborough, MA, USA; WA/10 mm). The load was recorded using a 100 N load cell, which was limited to 5 N effective range (HBM, Inc.; H2/100 N). The accuracy was in the order of 0.01 N. The displacement and the load signal were digitized using a data acquisition card (PCI-MIO-16E-4, National Instruments, Munich, Germany), yielding an accuracy of 0.08 N for the load signal and 0.06 mm for the strain signal. The sampling rate of the data was 10 Hz.

Adhesive strength was determined as the maximum shear force at rupture divided by the measured overlap area. Samples that failed to adhere, which became obvious during removal from the culture chamber or during placement into clamps, were assigned an adhesive strength of 0 kPa.

### Determination of glycosaminoglycan and collagen content

To assess the effects of the surface degradation treatment, the extracellular matrix content of cartilage blocks was analysed after being subjected to the respective agents. Additionally, the supernatant was analysed for released extracellular matrix components. Before analysis, cartilage samples were digested with 1 ml of a papainase solution (3.2 U/ml in buffer) for 18 h at 60°C. Sulfated GAG content was determined spectrophotometrically at 525 nm after the reaction with dimethylmethylene blue dye, using bovine chondroitin sulfate as standard [28]. Total collagen content was determined by measuring the amount of hydroxyproline according to [29], with some modifications. Digested sample (100 μl) was hydrolyzed with 100 μl 12 N hydrochloric acid for 16 h at 105°C. After hydrolysis, hydrochloric acid was evaporated. The dry samples were dissolved in 500 μl of bidistilled water. In a microtiter plate 100 μl of each sample were oxidized by 50 μl of a 0.05 M solution of chloramine T in a citrate buffer, pH 6, for 20 minutes. Afterwards, 50 μl of a 15% (mass/mass) dimethylaminobenzaldehyde solution in 4 mol perchloric acid in 70% isopropanol/water (mass/mass) was added and, after shaking, the plate was incubated for 30 minutes at 60°C. The plate was cooled down to room temperature and the absorbance of the samples was immediately measured at 557 nm using a microplate reader (CS-9301 PC, Shimadzu, Duisburg, Germany).

### Histology

Sample pairs were fixed in 2% glutaraldehyde and 4% formaldehyde in 0.1 M phosphate buffer, pH 7.3, for 30 minutes, and again fixed for 60 minutes in 4% formaldehyde, washed in buffer, embedded in Tissue Tek and frozen. Cryostat sections were cut perpendicular to the height of the articular cartilage block to a thickness of 5 μm and stained with toluidine blue for GAGs [5].

### Determination of cytotoxicity

The relative cytotoxicity of degrading and cross-linking reagents was tested by a resazurin reduction test obtained from Serotec Limited (Düsseldorf, Germany), which was used according to the manufacturer's instructions; a 10% resazurin solution was employed [30]. The oxidized, blue, non-fluorescent resazurin is reduced to a pink fluorescent dye in the medium by metabolic activity. For all tested degrading and cross-linking reagents, the same concentrations as stated in the cross-linking and surface degradation sections were used (see above).

### Statistical analysis

The statistical analysis was carried out using SPSS 12.0 G (SPSS, Munich, Germany). Of the free analysed parameters, adhesive strength (kPa), GAG content (μg), collagen content (μg), and relative fluorescence unit (RFU), the Kolmogorov-Smirnov test showed evidence against normal distribution for adhesive strength. Thus, the non-parametric Kruskal Wallis test for overall testing and the Mann-Whitney-U test for pairwise testing was applied to test for differences in adhesive strength between groups. For analysis of the GAG and collagen content of the cartilage blocks or cytotoxicity (RFU) of the applied agents, which were normally distributed, overall difference in the groups was assessed by analysis of variance (ANOVA) followed by *post hoc *comparisons made by Tukey's test. Throughout, statistical significance was accepted for *p *< 0.05.

## Results

### Adhesive strength of the bonding area

**Figure 3:**
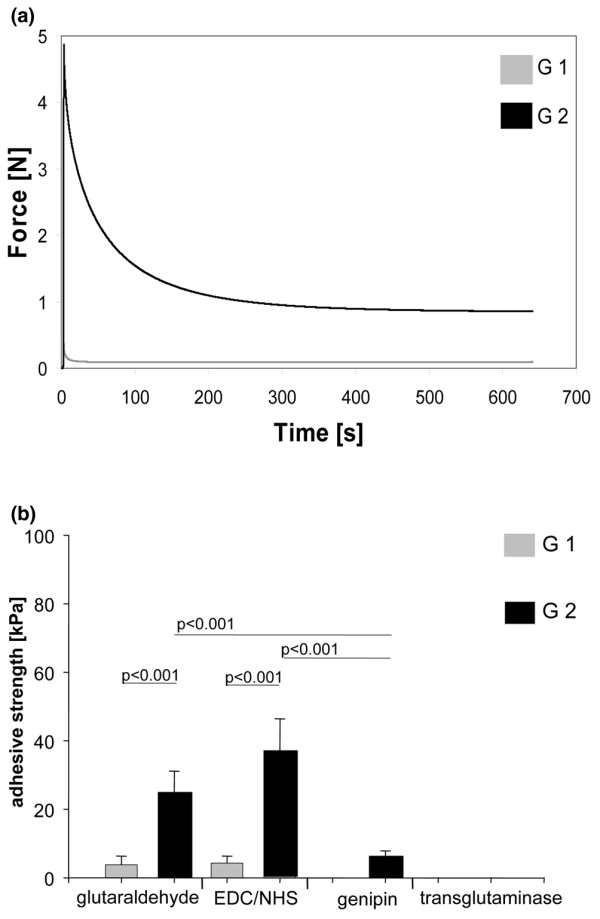
Stress-relaxation curves for the two sample geometries and bonding dependence on compression. **(a) **Stress-relaxation curves for the sample geometries G1 and G2 (see Figure 1e) were determined in a standardised creep modulus set-up. Samples were compressed by a stamp and the resulting force relaxation behaviour was analysed by recording the load over time. **(b) **G1 (almost no compression) or G2 (compression) cartilage blocks were subjected to different cross-linkers (without degrading pre-treatment). Adhesive strength as a measure of bonding was determined immediately after cross-linking. Bars represent the mean with standard error of the mean of at least 16 samples derived from 4 independent experiments, each with at least 4 replicates per group. *P *values in the graph are from pairwise comparisons using the Mann Whitney-U test. EDC, 1-ethyl-3-diaminopropyl-carbodiimide; NHS, N-hydroxysuccinimide.

At first, cartilage blocks of the two different geometries were fixed in partial apposition in the custom-made chamber. The relaxation behaviour of the G1 and G2 cartilage blocks is shown in Figure 3a. Due to the precise positioning of the G1 cartilage blocks in the chamber, immediately after mechanical fixation an instantaneous load drop to almost 0 N was observed. The remaining compressive load, in the order of 0.1 N, was attributed to swelling of the cartilage. In contrast, for the oversized G2 cartilage blocks, a compressive load of 5 N resulted from mechanical fixing by the stamp. With increasing incubation time and force relaxation, the load decreased and approached 1 N after approximately 400 s.

To compare the two geometries G1 and G2 with regard to cartilage bonding, the cross-linking agents were applied without prior surface degradation. The Kruskal Wallis test showed significant difference in adhesive strength (kPa) between the groups, which motivated us to perform pairwise testing. Compression to 83% of initial thickness during incubation (G2) resulted in strongly enhanced bonding after treatment with glutaraldehyde and EDC/NHS compared to no compression (Figure 3b). Using transglutaminase, no bonding occurred without prior surface degradation for either of the two geometries. Based on these results, the following experiments investigating the effects of the different combinations of degrading and cross-linking agents were conducted using G2 cartilage blocks. It should be noted that in experiments with neither surface degradation nor cross-linking or in experiments with surface degradation only, no bonding, for either G1 or G2, was achieved at all.

**Figure 4:**
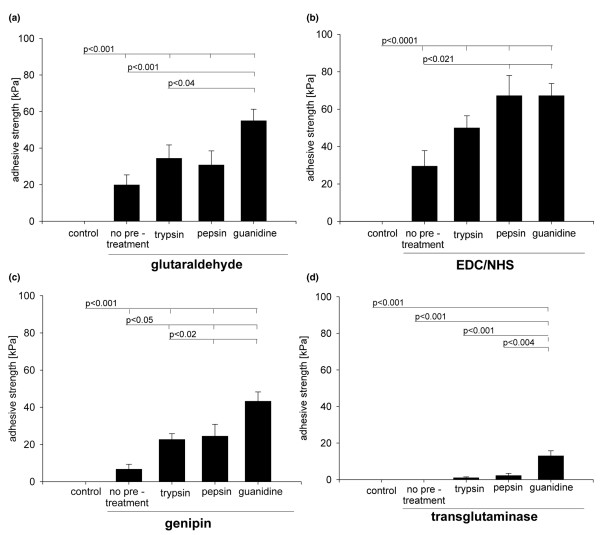
Bonding of cartilage blocks after treatment with surface-degrading reagents and subsequent cross-linking. Adhesive strength as a measure of bonding was determined immediately after cross-linking with **(a) **glutaraldehyde, **(b) **1-Ethyl-3-diaminopropyl-carbodiimide (EDC)/N-hydroxysuccinimide (NHS), **(c) **genipin, or **(d) **transglutaminase. Before cross-linking, cartilage blocks were pre-treated with either trypsin, pepsin, or guanidine, or blocks were cross-linked without pre-treatment ('no pre-treatment'). In the control group, neither pre-treatment nor cross-linking were performed. Bars represent the mean with standard error of the mean of 20 samples derived from 4 independent experiments, each with 5 replicates per group. *P *values are from Mann Whitney-U test for pairwise comparisons.

The four cross-linking reagents glutaraldehyde, genipin, EDC/NHS, and transglutaminase were each combined with the pre-treatments trypsin, pepsin, and guanidine; the resulting bonding quality measured as adhesive strength is shown in Figure 4. The Kruskal Wallis test showed significant differences in adhesive strength (kPa) between the groups, prompting us to perform pairwise testing. With glutaraldehyde, only guanidine pre-treatment led to a significant increase of adhesive strength (55 kPa compared to 20 kPa for the group with no pre-treatment; Figure 4a). In combination with EDC/NHS, pepsin or guanidine pre-treatment increased the adhesive strength to 65 kPa, exhibiting the highest values seen among all combinations of pre-treatment and cross-linking reagents in this study (30 kPa for no pre-treatment; Figure 4b). With genipin, all three pre-treatments led to a significantly increased adhesive strength, with the highest values detected with guanidine; however, mean values were all below those for EDC/NHS and glutaraldehyde (Figure 4c). For transglutaminase cross-linking, pre-treatment with guanidine was necessary to induce noticeable bonding. Overall, transglutaminase clearly resulted in the lowest values for adhesive strength compared to all other cross-linkers (Figure 4d).

### Effects of surface degradation on glycosaminoglycan and collagen content

**Figure 5:**
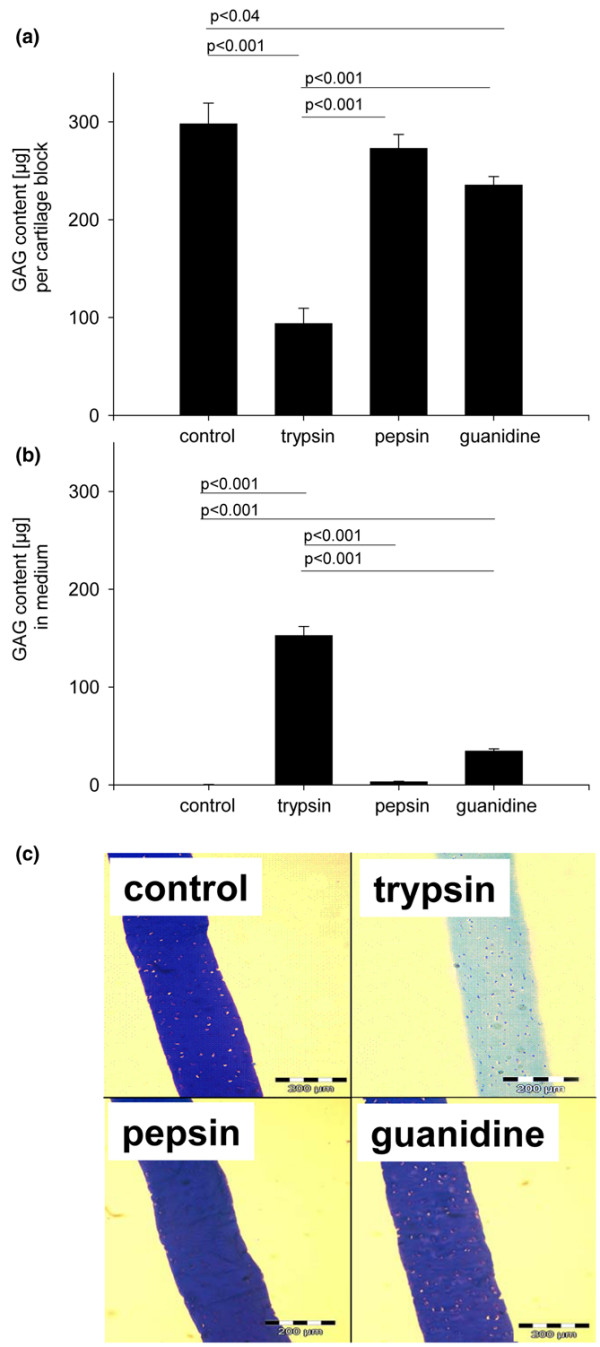
Glycosaminoglycan (GAG) release from cartilage blocks determined after treatment with surface-degrading agents. Cartilage blocks were subjected to pre-treatment with trypsin, pepsin, or guanidine, as indicated for the bonding experiments; the samples in the control group were incubated in PBS buffer. **(a) **Subsequently, the GAG content within the cartilage blocks was determined. **(b) **Additionally, the amount of GAG released into the medium (per cartilage block) was measured. Nine samples were measured per group. Bars represent the mean with standard error of the mean. *P *values are from *post hoc *Tukey test for pairwise comparisons. **(c) **Additionally, histological cross-sections of cartilage blocks were stained for GAGs.

To determine the effects of the degrading agents on extracellular matrix content, the GAG and total collagen content were determined in cartilage samples and the respective supernatants after treatment. The ANOVA test showed significant differences between the groups (*p *< 0.001). Trypsin treatment strongly decreased the GAG content of cartilage samples to 31% of the control group (Figure 5a); whereas untreated control blocks had a GAG content of 4.2% per wet weight, trypsin reduced the GAG content to 1.3%. Guanidine treatment resulted in a reduction in GAG content to 78% of that of the control group, whereas only small amounts of GAG were released from the cartilage samples treated with pepsin (Figure 5a). The GAG release from cartilage samples was confirmed by analysis of the corresponding supernatants (Figure 5b) and staining of histological cross-sections of cartilage blocks with toluidine blue for GAG (Figure 5c). The collagen content of untreated control blocks was 12.8% per wet weight. For all three treatments, trypsin, pepsin, or guanidine, no significant reduction in collagen content in the cartilage samples was detected.

### Relative cytotoxicity

**Figure 6:**
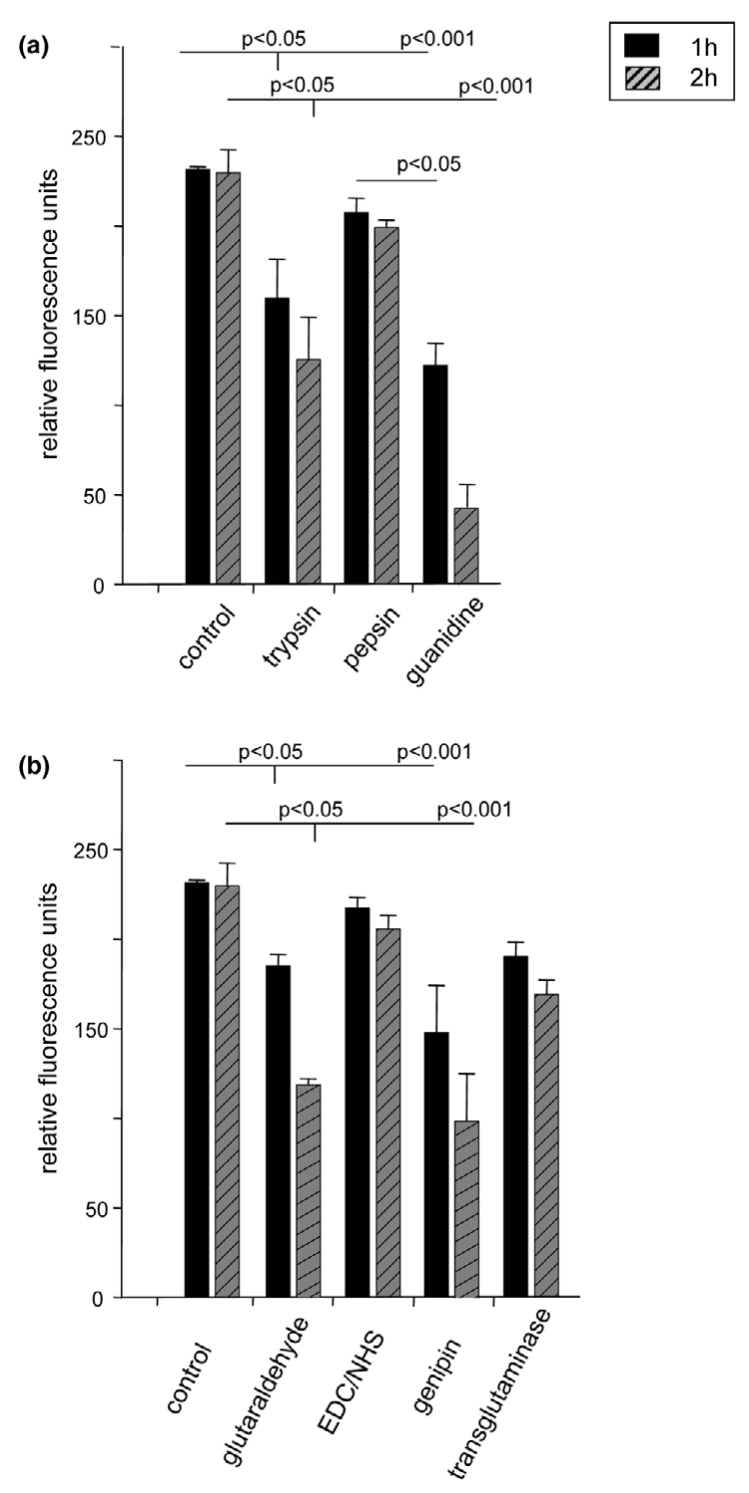
Cytotoxicity of degrading and cross-linking agents. The relative cytotoxicity of **(a) **degrading and **(b) **cross-linking agents was determined using the resazurin assay (expressed as relative fluorescence units). Cartilage blocks were treated with the respective reagents for either one or two hours. The controls were incubated in PBS. *P *values are from Tukey test for pairwise comparisons. EDC, 1-ethyl-3-diaminopropyl-carbodiimide; NHS, N-hydroxysuccinimide.

The resazurin cytotoxicity test performed on cartilage samples revealed distinct differences for the reagents employed in this study (*p *value from ANOVA, *p *< 0.001). Among the degrading reagents, pepsin had only small cytotoxic effects whereas guanidine exhibited strong effects in comparison to the non-treated control group (Figure 6a). In the cross-linking reagent group, EDC/NHS showed almost no effect, whereas reduced metabolic activity to less than 50% within 2 h was detected for glutaraldehyde and genipin (Figure 6b).

## Discussion

In this study, the effects of compression of the cartilage interface, surface degradation, and biochemical cross-linking on articular cartilage bonding were investigated. Specific emphasis was put on the resulting mechanical stability of the bonded interface due to molecular bridging of opposing surface structures. This immediate repair technique might provide one further option for the therapeutic treatment of articular cartilage wounds.

### Compression

In the absence of treatment with cross-linking agents, neither just laying the cartilage blocks together nor compressing them (to 83% of initial thickness) had any effect on bonding between them. Although the cross-linking reagents EDC/NHS and glutaraldehyde led to measurable bonding of cartilage blocks in the absence of compressive strain, large increases in adhesive strength were achieved by additional compressive load during the bonding procedure. Therefore, all experiments investigating combinations of cross-linking reagents with a pre-treatment with surface degrading agents were carried out under compressive load conditions.

### Cross-linking

EDC/NHS can non-specifically catalyse covalent binding of the amino or carboxyl groups of collagen; furthermore, carboxylic groups of GAGs may also be involved. In our study, EDC/NHS was the best cross-linking reagent with regard to bonding of articular cartilage blocks in all investigations, that is, when comparing the cross-linkers alone and in combination with degrading pre-treatment. The combinations of EDC/NHS with pepsin or guanidine pre-treatment led to the highest adhesive strengths detected in this study. To date, cell-based articular cartilage repair *in vitro *has been correlated with cell metabolism [2], collagen deposition [1] and collagen cross-linking [3,31]. However, the impressive results yielded with EDC/NHS, the only cross-linker in this study able to additionally catalyse binding of GAGs, may support the idea that molecules other than those involved in the collagen network can also contribute to the integrative process *in vitro*.

With regard to cytoxicity, even after exposure for two hours, EDC/NHS elicited no significant effects, whereas glutaraldehyde and genipin compromised cell vitality considerably. In previous investigations, EDC/NHS has also been observed to be advantageous in this respect, compared to glutaraldehyde [32]. Furthermore, the presented adhesive strength data resulted from a total incubation with cross-linking reagents for two hours. In additional experiments in which guanidine or pepsin pre-treated samples were exposed to EDC/NHS for only 30 or 10 minutes, an adhesive strength between 58 and 56 kPa was observed, that is, there was no significant difference to the prolonged treatment of two hours. An exposure for only 10 minutes would further reduce the risk of any cytotoxic effects.

Glutaraldehyde and genipin cross-link amino groups of proteins; glutaraldehyde molecules can cross-link to each other and the chain building properties may have beneficial effects in comparison to genipin, which can only cross-link in pairs [33]. In this study, glutaraldehyde alone yielded higher adhesive strengths than genipin alone and also had slight advantages in combination with pre-treatments. With regard to cytotoxicity, a better tolerance for genipin in comparison to glutaraldehyde has been shown in other investigations utilising 3T3 mouse fibroblasts [34], human osteoblasts [35] and a subcutaneous chamber in mice [36]. In our study, both agents exhibited similarly strong cytotoxic effects.

Transglutaminase, a naturally occurring enzyme in articular cartilage, catalyses a specific collagen cross-linking reaction between lysine and glutamine residues. This enzyme has been previously introduced, combined with compressive load, to enhance integrative bonding of articular cartilage wounds [26]. In our investigation, bonding was detectable only in combination with guanidine pre-treatment; however, compared to the other cross-linking options investigated in this study, transglutaminase resulted in rather weak bonding. The protocol employed in this study was initially described by Chen and colleagues [27] for cross-linking collagen matrices and may not be well transferable to articular cartilage. The reduced reaction time in this study (2 h) compared to that reported previously (12 h) may also have contributed to the reduced effect. Nevertheless, transglutaminase may still play an important role in *in vitro *and *in vivo *integrative repair. Transglutaminase has been previously shown to be biocompatible [37], which was also found in this study, with no significant differences to the control. This enzyme, with its specific catalysing mechanism, may be especially beneficial in an ongoing integrative repair process in which newly synthesised collagen fibrils are present, in contrast to a static experimental setting such as used in this study.

For many years, soft tissue adhesives like fibrin [38] have been used for cartilage repair or as an additive in autologous chondrocyte transplantation [39,40]. They have been found to be supportive in chondrocyte transplantation or to seal the periosteum flap to the cartilage *in vitro*, but *in vivo *fibrin glue did not provide enough mechanical strength to hold the periosteum flap in place [41]. As a further alternative, several synthetic materials have been employed as glues for soft tissues, for example, aminopropyltrimethoxysilane-methylenebisacrylamide siloxane or n-butylcyanoacrylate [42]. In general, the bonding mechanisms of these polymeric substances differ from those of the chemical reagents used in the present work. The polymers penetrate the soft tissue to a certain extent and adhesion is achieved through an interpenetrating network that is irremovable and may impair tissue development at the integration site. In contrast, the chemical cross-linker induces formation of covalent bonds on the surface of the soft tissue. In our opinion, EDC/NHS may be beneficial compared to polymer glue and other chemical cross-linkers (glutaraldehyde and genipin) due to its pure catalysing function. EDC/NHS will not be incorporated into the cartilage and can be easily removed and the scar tissue can be remodelled by cell and extracellular matrix turnover.

### Degradation

Degradation or swelling of articular cartilage surfaces have been reported to be beneficial in cell-based integrative repair *in vitro *[6,9-12]. In our study, bonding between cartilage blocks did not occur by merely treating the blocks with trypsin, pepsin, or guanidine (even under compressive conditions). However, pre-treatment with the endopeptidases pepsin or trypsin before cross-linking led to distinct improvements in bonding compared to the use of cross-linkers alone. This was particularly the case for the combination of pepsin with EDC/NHS, for which high values for adhesive strength were achieved. With regard to cytoxicity, pepsin led to no significant effects, whereas trypsin treatment compromised cell vitality considerably.

Pre-treatment with guanidine led to the highest adhesive strengths in combination with all cross-linkers compared to the endopeptidase pre-treatments. Unfortunately, guanidine elicited the strongest cytotoxic effects of all reagents in the study. It is noteworthy that the achieved mechanical bonding is comparable to previous studies employing a similar model. Reindel and colleagues [4] first reported a mechanical adhesive strength of 34 kPa in integrative experiments. Subsequently, studies including degradation with trypsin followed by cultivation reported enhanced adhesive strengths up to 100 kPa [6]. In the present study, adhesive strengths up to 65 kPa were achieved by guanidine or pepsin pre-treatment and EDC/NHS cross-linking.

Previously, it was assumed that degrading surface treatment led to cell proliferation or stimulation of cell metabolism [12]. The observation from our investigations that cross-linking reagents lead to significantly stronger bonding of cartilage blocks after degradation or swelling pre-treatment implies another hypothesis. The accessibility of functional groups is enhanced by both treatments and, therefore, may have led to a better bonding in our study and better integrative repair in previously described studies. It remains to be clarified on which components of the extracellular matrix these functional groups are located. The endopeptidase trypsin was clearly the most effective at releasing GAGs from the cartilage blocks, whereas pepsin released only a minor fraction of the GAGs. The treatment of cartilage blocks with guanidine prior to biochemical cross-linking leads, in theory, primarily to reduction of non-covalent bonding between molecules of the extracellular matrix. The tertiary structure of matrix molecules, especially GAGs, becomes more open after hydrogen bonds are broken. Additionally, elevated water uptake may occur due to more accessible functional groups or a loosened collagen network with increased pore diameter (swelling). Nevertheless, in our study, guanidine also led to the release of a significant fraction of GAGs from the cartilage blocks. As trypsin led to the lowest adhesive strength values of all surface-degrading agents in this study, the bonding observed can not be directly correlated with the amounts of GAGs released. On the contrary, the large amount of GAGs released by trypsin may have compromised the cartilage structure. For total collagen, no significant release was detected for all the pre-treatments. However, only small changes in the structure of the cartilage surface, which are triggered by the pre-treatment agents, but which are not detectable by the assays employed in this study, may be necessary to elicit distinctly improved responses to the cross-linkers.

Clarification of the mechanisms involved appears to be a worthwhile subject for further investigation. Future studies should also address the fact that immature and mature cartilage differ in extracellular matrix content, structure and mechanical properties [43-45]. In aging, cartilage undergoes structural changes that affect the susceptibility to degradation [46-48]. It is also known that integrative bonding is influenced by the developmental stage of articular cartilage [4]. Therefore, in future studies, the introduced treatment may have to be adjusted to adult cartilage. Furthermore, it has to be noted that for clinical applications special care should be taken to limit any treatment with degrading and cross-linking agents to the area close to the cartilage wound surface. In addition, cell culture experiments after bonding should assure the long-term viability of the treated cartilage.

## Conclusion

This study clearly demonstrates that immediate bonding of articular cartilage blocks can be achieved by means of chemical cross-linking. Adhesive strength was superior under compressive conditions compared to no compression. In general, pre-treatment with surface-degrading enzymes or swelling by guanidine salt led to distinct enhancement of cartilage bonding after chemical cross-linking. Taking both the observed bonding and the cell vitality after treatment into account, the combination of pepsin pre-treatment and cross-linking with EDC/NHS appears to be the most favourable with regard to this study. The presented work suggests that a combination of selected surface-degrading agents and chemical cross-linkers is a promising option for enhancing bonding of opposed surfaces in cartilage repair.

## Abbreviations

EDC = 1-ethyl-3-diaminopropyl-carbodiimide; G = geometry (of cartilage blocks); GAG = glycosaminoglycan; NHS = N-hydroxysuccinimide; PBS = phosphate-buffered saline; RFU = relative fluorescence unit.

## Competing interests

The authors declare that they have no competing interests.

## Authors' contributions

CE conceived of the study and its design, participated in the bonding experiments, and helped to draft the manuscript. TB participated in the design of the study and drafted the manuscript. RM participated in the design of the study, specifically with regard to chemical cross-linking. SSvG carried out the bonding experiments and the cytotoxicity assay. JB carried out the analysis of the extracellular matrix content. JF carried out the analysis of the relaxation behaviour and participated in the bonding experiments. IH helped to perform the statistical analysis. MN and JH participated in the design and coordination of the study. All authors read and approved the final manuscript.
